# Case of Rupture of the Uterus, Successfully Treated

**Published:** 1830-11

**Authors:** Wm. S. Hendrie

**Affiliations:** Hilltown, Penn.


RUPTURE OF THE UTERUS.

*Case of Rupture of the Uterus, successfully treated.* By Wm. S. Hendrie, m.d. of Hilltown, Penn.*

Mrs. ——, a small woman, of healthy constitution, thirty-three years of age, pregnant with her eleventh child, was, on the 17th of May, 1829, at about twelve o'clock in the morning, taken with labour pains, and sent for her physician, Dr. Samuel Carey. Her labours had been uniformly tedious and painful; rendered so in a great measure by a small bony tumor, situated on the projection of the sacrum, lessening the anterior-posterior diameter of the pelvis, at least one inch or one inch and a half.† At ten o'clock the os uteri was so high as to be reached with difficulty by a common examination: it was, however, considerably dilated, and the presentation natural. She was bled to the amount of twenty ounces, and an enema administered, by which the rectum was freely evacuated. As had been the practice with most of her preceding labours, the Secale cornutum was given: this soon produced its usual effect, and continued for some time. During a violent expulsive effort, the pains suddenly ceased, the abdominal tumor changed its regular appearance, she declared something unusual had occurred, and supposed she was about to be delivered of twins: the head immediately receded, and considerable hemorrhage took place.

This is the history I received of the case from Dr. Carey. I saw her at nine o'clock in the evening, about eight hours after the accident had occurred. At this time she was labouring under great anxiety; considerable prostration; pulse frequent and feeble; extremities cold; abdomen very much distended, and in some parts extremely tender; hemorrhage very much diminished: the child could be distinctly traced through the abdominal parietes. No doubt existed with regard to the nature of the accident that had occurred. After a careful examination, we ascertained that the rupture was on the anterior part of the uterus; that the child, together with the placenta, had entirely escaped into the cavity of the abdomen: the rent commenced in the cervix, on the right side, extended obliquely toward the left about three inches; the remainder of the laceration was at the junction of the uterus with the vagina. The fundus of the uterus was pretty firmly contracted, but the

* American Journal of Med. Sciences.

† Of the ten preceding labours, the forceps had been used twice: she has, however, given birth to five living children.

body and cervix flaccid and yielding. The child lay obliquely across the abdomen, the head towards the right iliac region.

Notwithstanding the length of time that had elapsed since the occurrence of the accident, as the parts concerned in the injury did not oppose any obstacle to delivery, *per vias naturales,* we felt justified in making that attempt, especially as the history of all preceding cases left us nothing to hope from the conservative powers of nature. Having made a candid statement of the probable result to our patient and her friends, they promptly acquiesced, and even expressed a willingness to submit to any operation we should think necessary, to relieve her present anxiety and distress.

Having placed her in a favorable position, the hand was introduced, the bladder being previously evacuated: the feet were readily obtained, and the body of the child delivered with the utmost facility. Some difficulty was experienced in getting the head through the superior strait of the pelvis, owing to the exostosis already mentioned: this was finally accomplished with the aid of the crochet. The hand was again introduced, for the purpose of extracting the placenta, which was found in the left side of the abdomen. Some large coagula were brought away entangled with the secundines. In searching for the placenta, my hand was in contact with the naked intestines; from which circumstance no doubt can remain of the rupture being complete. After ascertaining that none of the bowels protruded from the laceration, she was placed in bed, in a very exhausted condition; a cordial anodyne was given. Two hours afterwards she expressed herself as feeling quite comfortable.

18th, six o'clock A.M. Has rested very well since twelve o'clock. System appears to be reacting; pulse remains small and frequent; complains of some soreness, but no pain, yet the abdomen is quite tender to the touch, and is somewhat distended. Directed fomentations to be applied to the abdomen, and a cathartic mixture.

Six o'clock P.M. Abdomen greatly distended, and extremely painful, exquisitely so on pressure; pulse frequent and tense; skin dry and hot, cathartic has not operated. Ordered V.S. ℥xviij.; cathartic medicine to be repeated, and its operation solicited by an occasional enema; fomentations continued.

19th. Passed a very restless night: bowels freely opened this morning, since which the pain and distention of the abdomen very much diminished; pulse less frequent, yet somewhat tense. Ordered venesection ℥vij.; Nitro-antimon. pulv. every two hours.

21st. Is much better; no pain, and very little tenderness on pressure; pulse soft; skin cool; countenance lively and cheerful.

21st et seq. She is still improving; thinks herself able, if permitted, to sit up and have her bed adjusted. Rest, and a low diet, were enjoined, and persevered in for several days; her convalescence was rapid and uninterrupted: four weeks after the accident, she was able to attend to her domestic affairs; complains of no particular uneasiness, except the inconvenience resulting from a urinary fistula.

January 20th, 1830. Enjoys at present remarkably good health; has menstruated regularly since August. In July an apparatus was procured, for the purpose of obviating the inconvenience arising from the fistulous opening in the bladder: it has been worn till within the last few weeks, and has affected a radical cure.

---

